# A Three-step Etch-and-Rinse vs a Universal Adhesive in Nanohybrid Composite Anterior Restorations: A Retrospective Clinical Evaluation

**DOI:** 10.3290/j.jad.b4043039

**Published:** 2023-04-24

**Authors:** Allegra Comba, Andrea Baldi, Massimo Carossa, Gaetano Paolone, Ilaria Stura, Giuseppe Migliaretti, Nicola Scotti

**Affiliations:** a Assistant Professor, Department of Surgical Sciences, Dental School, University of Turin, Turin, Italy. Wrote the manuscript.; b PhD Student, Department of Surgical Sciences, Dental School, University of Turin, Turin, Italy. Patient recall management and evaluation.; c PhD, Clinical Tutor, Department of Surgical Sciences, Dental School, University of Turin, Turin, Italy. Revised the manuscript.; d PhD, Department of Dentistry, IRCCS San Raffaele Hospital and Dental School, Vita Salute University, Milan, Italy. Revised the manuscript.; e Research Fellow, Department of Neurosciences “Rita Levi Montalcini”, University of Turin, Turin, Italy. Statistical analysis.; f Associate Professor, Department of Public Health and Pediatric Sciences, University of Turin, Turin, Italy. Statistical analysis.; g Associate Professor, Department of Surgical Sciences, Dental School, University of Turin, Turin, Italy. Coordinator of the research project and patient treatment.

**Keywords:** anterior teeth, Class-IV, direct restoration, etch-and-rinse, universal adhesive.

## Abstract

**Purpose:**

To retrospectively evaluate the clinical behavior of direct anterior composite restorations performed with a universal adhesive or with a three-step etch-and-rinse (E&R) adhesive.

**Material and Methods:**

Patients were randomly treated with a three-step E&R adhesive (Optibond FL, Kerr) or a universal adhesive (Clearfil Universal Bond Quick, Kuraray Noritake) applied in E&R mode. All restorations were performed with a nanohybrid composite (ClearFil Majesty ES-2, Kuraray Noritake) by the same experienced operator. Two calibrated examiners evaluated the restorations using a dental mirror and explorer, in accordance with modified United States Public Health Service (USPHS) procedures. Clinical events were registered and classified as either failure (F), survival (SR), or success (S).

**Results:**

168 restorations were evaluated in 90 patients with an average follow-up period of 37.9 (± 22.9) months. A total of 132 restorations were performed on vital teeth, and 36 were performed on endodontically treated teeth (ETT). A total of 128 Class-IV and 40 Class-III restorations were performed. In 89 restorations, a three-step E&R adhesive was applied (14 Class-III and 75 Class-IV), while in 79, a universal adhesive was used (26 Class-III and 53 Class-IV, p = 0.0091). A Cox regression analysis was performed (p < 0.05) to analyze which factors were involved in the failure of the restorations, considering failure (F) as restorations that needed re-intervention at the follow-up period of 37.9 (± 22.9) months. No statistically significant differences were observed when considering parameters directly involved with the adhesives tested. Endodontically treated teeth were more prone to fractures (p = 0.0006) compared to vital teeth. Restorations made with universal adhesives failed by fracturing significantly more frequently (p = 0.0234), while restorations made on endodontically treated teeth had a significantly worse outcome (p = 0.0001). Restorations made on canines also failed significantly more frequently (HR = 3.8, 95% CI = 1.4–10.1, p = 0.0062).

**Conclusions:**

Based on the obtained results, both the universal adhesive and the three-step E&R adhesive proved to be good treatment choices for direct anterior restorations after 37.9 (± 22.9) months of follow-up. Tooth vitality seems fundamental for the prognosis of a direct anterior composite restoration over time.

Considering posterior teeth, several studies have evaluated the longevity and compared the properties of direct nanofilled-composite restorations.^10^ In 2015, Beck et al^2^ published a meta-analysis with a follow-up period of 19 years, observing that the main short-term causes of failure were fractures of the restorations, secondary caries, and marginal gaps, while in the long-term assessment, material fracture and secondary caries were similarly distributed. Similar conclusions were reported by Alvanforoush et al^1^ in a recently published study. Regarding anterior teeth, Heinze et al^22^ published a meta-analysis on the efficacy of composite resin restorations, observing a mean overall success rate (without replacement) of 95% for Class-III and 90% for Class-IV restorations after 10 years. However, the long-term success of anterior direct restorations is influenced by several factors, such as restoration form, shade selection, marginal integrity, and surface texture. These factors are very important in the esthetic appearance and the medium- to long-term outcome of an anterior tooth restored with a composite.^36^ Indeed, Demarco et al^11^ pointed out that tooth/restoration fractures were the most common reason for anterior composite restoration failure, while esthetic failure was more frequent when restorations were essentially placed for esthetic reasons. However, Lambert^28^ observed that the use of direct composite resin restorations offers the dentist the easiest and most economical way to create an esthetic change in the smiles of teenagers and young adults, with conservative and functional restorations that present excellent longevity.

The spread and success of resin composites, even in anterior teeth, are inextricably linked to the development of increasingly effective adhesives. The interaction between adhesives and dental substrates is based on two different modes: etch-and-rinse (E&R) and self-etch (SE).^49^ In E&R adhesives,^37^ both enamel and dentin are conditioned with 35%–37% phosphoric acid in a dedicated step before applying the primer, and the bonding resin is applied separately (three steps) or as a single-bottle formulation (two steps). From a clinical point of view, three-step E&R adhesives demonstrate better performance in-vitro and in-vivo than do two-step adhesives.^13,23^ On the other hand, with SE adhesives, a dedicated etching step of both enamel and dentin is not required before the application of the self-etching primer and the adhesive resin, which can be provided separately (two steps) or together in a single bottle (one step).^45,50^ However, selective etching of the enamel for 15 s has proven to be clinically successful in providing better long-term enamel bonding stability, and is usually recommended.^39^

A recent development in adhesive science is represented by universal adhesives, a single-bottle solution that can be used in both E&R and SE modes.^43^ As mentioned above, several studies suggested the use of phosphoric acid on enamel margins for 10–15 s to achieve better long-term clinical results in terms of marginal integrity and prevention of marginal discoloration.^21,33,52^ Universal adhesives can be optionally applied with phosphoric acid in the entire cavity due to the intrinsic acidity of interacting with the hard tissues of teeth without etching. When used in SE mode, ie, without dentin etching, the different functional monomers of universal adhesives establish a very stable chemical bond with the calcium and phosphate ions of the hydroxyapatite. However, the strong micromechanical interlocking is partially lost, which is fundamental when universal adhesives are employed in E&R mode. Therefore, with dentin pre-etching, the adhesive solution can infiltrate the exposed collagen-fiber network, thus establishing micromechanical interlocking. This one-bottle approach to treating dental tissues undoubtedly simplifies adhesive procedures. However, due to their recent introduction, studies of their applications in the treatment of direct anterior and posterior restorations are still few and lack sufficient follow-up periods to establish their effectiveness.^4^ Longer follow-up studies are limited to the treatment of non-carious cervical lesions (NCCL).^14,32,41^

Another fundamental parameter to take into consideration before proceeding with restoration is tooth vitality. Endodontically treated teeth (ETT) present several mechanical alterations, such as greater fragility, mainly due to the loss of tooth structure.^25,27,40^

Furthermore, anterior teeth are subjected to high extra-axial forces during protrusion and lateral movements, making them more susceptible to biomechanical failure. However, modern minimally invasive dentistry has progessed to more conservative techniques, and direct restorations have been proposed to restore ETT given sufficient healthy dental hard tissue and cervical enamel.^30,42^ The advantages of conservative preparations include the reinforcement of residual dental tissues, reparability, and good esthetics at low costs. In 2013, Paolone et al^35^ analyzed several clinical cases involving restoration of anterior ETT and concluded that direct restorations could lead to successful results. Similar conclusions were drawn by Von Stein-Lausnitz et al,^51^ who demonstrated how Class-III cavities might be successfully restored with direct composite restorations.

To the best of our knowledge, despite the widespread use of composite materials in the anterior area, there is a paucity of long-term clinical studies regarding their use in combination with different adhesives. Therefore, the aim of the present retrospective evaluation was to compare the clinical performance of direct Class-III and IV composite restorations applied with a universal adhesive.

The null hypotheses tested were that there are no significant differences in terms of clinical performance between 1. universal and three-step adhesives applied in E&R mode and 2. ETT and vital teeth.

## Materials and Methods

### Study Characteristics, Participants, and Design

This retrospective clinical study was conducted at the Department of Cariology and Operative Dentistry, University of Turin. All patients who received direct anterior restorations in the anterior maxillary teeth by the last author were selected for this retrospective analysis. All patients were contacted by telephone or mail. Those patients who were able to participate in the study signed a written informed consent form prior to the start of the clinical evaluation. This retrospective protocol was conducted in accordance with the recommendations of the Declaration of Helsinki, as revised in Fortaleza, Brazil and adopted on 19th October 2013, for investigations with human subjects. The ethics committee of CIR Dental School – Lingotto (University of Turin) approved the study protocol (DS_00093_2018).

### Sample Size Estimation

Considering a minimum success of 89% and a significant difference of 11%, the a priori sample size of 90 patients per group was chosen to reach a power of 81%.^26^ The calculation was done by SAS Statistics Software v. 9.4 (Cary, NC, USA).

### Inclusion and Exclusion Criteria

For this retrospective clinical evaluation, patients meeting the following inclusion criteria were recruited: no systemic disease, age between 14 and 40 years, good oral hygiene (full-mouth plaque score < 20%), no active periodontal or pulpal disease, occlusal stability, and maxillary, mandibular, and canine restorative treatment performed for different reasons (primary and secondary caries, trauma, esthetics, and fractures).

Exclusion criteria were systemic disease, uncontrolled parafunction, reduced dimension of vertical occlusion, insufficient oral hygiene leading to multiple caries, periodontal and gingival disease, and absence of antagonist teeth. All selected restorations were performed with the same nanohybrid composite (Clearfil Majesty ES-2, Kuraray Noritake; Tokyo, Japan) by the same experienced operator. With regard to the adhesive approach, patients were randomly treated with a three-step E&R adhesive (Optibond FL, Kerr; Orange, USA) or a universal adhesive (Clearfil Universal Bond Quick, Kuraray, Tokyo, Japan) applied in E&R mode.

### Restorative Procedure

Patients with the above-mentioned parameters underwent an oral hygiene session for plaque and calculus removal. Diagnostic models were obtained by taking maxillary and mandibular impressions, and a wax-up was then made to create a silicone guide for clinical procedures.

All restorations were performed following a standardized procedure according to the manufacturer’s instructions: color selection through a personalized shade guide, rubber-dam placement to isolate the anterior region, tooth-surface cleaning using pumice, cavity preparation after removal of any carious tissue, and beveling of buccal enamel margins with extra-fine–grit diamond burs.

The patients were randomly divided into two groups based on the adhesive approach used:

- Group 1: Three-step E&R (Optibond FL, Kerr). 30-40 s etching with 37% phosphoric acid on enamel and 10-15 s on dentin, generous water rinsing for 30 s followed by drying, primer application and evaporation, application of bonding resin, and 20- to 40-s light curing with a polywave LED unit according to the number of restorations performed (Valo, Ultradent; South Jordan, UT, USA).
- Group 2: Universal adhesive in E&R mode (Clearfil Universal Bond Quick, Kuraray). 10-15 s etching with 37% phosphoric acid on enamel and dentin, generous water rinsing for 30 s followed by drying, application of bonding resin for 15-20 s, and 20- to 40-s light curing with a polywave LED unit according to the number of restorations performed (Valo, Ultradent).

Restorations were performed as follows: composite layering using a silicon index and a transparent silicon matrix with a natural layering technique, applying a hydrophobic coating and 20-s light curing, contouring and finishing with a flame coarse- to fine-grit diamond bur (8859.314.014. 8368.204.023 Komet, Gebr Brasseler; Lemgo, Germany) and abrasive disks with decreasing grain size (Sof-Lex 3M Oral Care; St Paul, MN, USA) in a low-speed handpiece, removing rubber-dam, polishing and finishing procedures with an auto-polishing brush and felt disks. After completing restoration placement, patients were informed about oral hygiene measures for cleaning the new restorations with a toothbrush and dental floss. After one week, a chromatic evaluation was performed to check the final esthetic result, and any necessary shade corrections were performed. Intraoral photographs were then taken to support further evaluation at baseline and at each control appointment.

### Evaluation Procedure

The restorations were evaluated between February and July 2019 by two blinded, calibrated examiners using a dental mirror and explorer in accordance with the modified USPHS criteria, as first described by Cvar and Ryge,^8^ adapted by Wilson et al,^54^ and further revised by Lempel et al.^29^ The dentists were trained and calibrated before the start of the evaluation. Cohen’s kappa statistic was used to calculate observer agreement. This study found excellent intraobserver (kappa values of 0.78 and 0.80) and interobserver (kappa value of 0.80) agreement.

### Definition of Clinical Event

**Fig 1:**
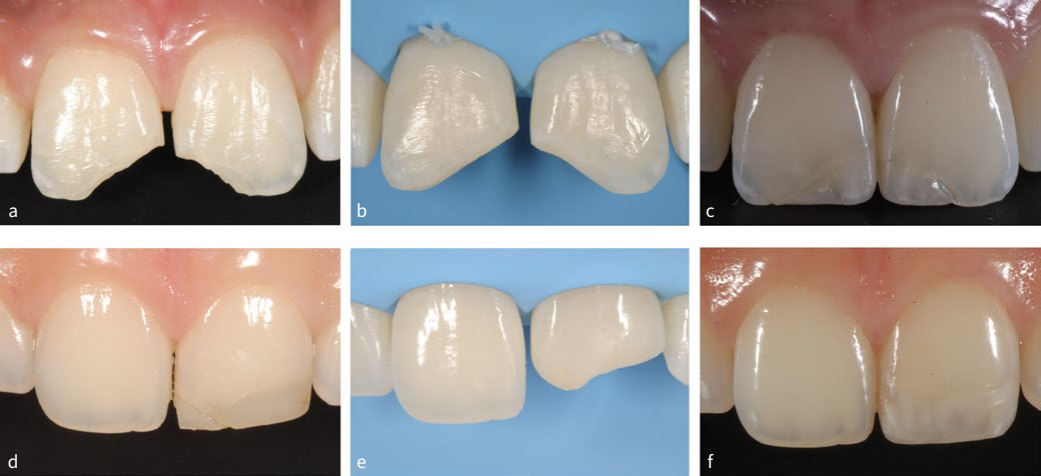
Example of clinical cases selected for the retrospective study (follow-up period of 58.7 months) with failure and survival of the restorations placed: a) initial pre-operative view; b) intra-operative view of the prepared cavity; c) failure of the restoration at 58.7 months with fracture of the composite material; d) initial pre-operative view; e) intra-operative picture of the prepared cavity; f) survival of the restoration at 42.1 months with marginal discoloration, surface roughness and color match.

**Fig 2:**
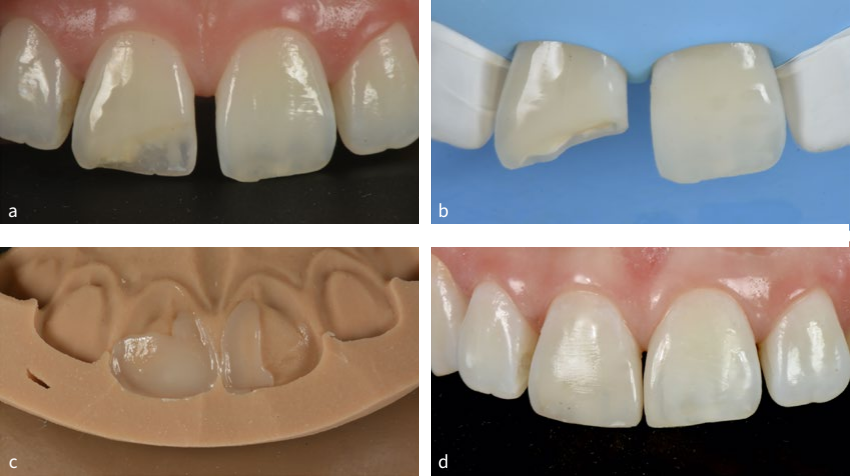
Example of a clinical case selected for the retrospective study (follow-up period of 36.3 months) rated as successful: a) initial pre-operative view of the initial case before treatment; b) intra-operative view of the prepared cavity; c) silicon index for palatal wall reconstruction; d) success of the restoration at 36.3 months.

Failure (F) was registered when the restoration was deeply infiltrated, fractured, or lost, making repair impossible. Survival (SR) was registered when reparable, less damaging events occurred, such as minor composite fractures, chipping, small marginal gaps, or color/surface deterioration (Figs 1 and 2). In such cases, restorations were repaired with additional composite after sandblasting the surface with 50-µm alumina oxide and application of silane and adhesive, or the surface was re-finished to recreate texture or re-polished. The type of unfavorable event was registered in the patients’ record. Restorations with no failure or unfavorable events were classified as successful (S).

### Statistical Analysis

Descriptive statistics were performed, and categorical variables were compared between the groups using the chi-squared test to estimate the association between vital and non-vital teeth as well as the three-step E&R adhesive and the universal adhesive employed in E&R mode. The level of significance was 5% (p < 0.05), and the data were analyzed with SAS 9.4 software.

Restoration characteristics, including the number of unacceptable restorations, failures, and complications, were described with descriptive statistics using percentages of the overall number of samples. To understand which factors were involved in the failure of restorations, Cox regression analysis was performed. The variables considered were the adhesive employed, tooth element, type of restoration, and vitality of the tooth. The results are expressed as a hazard ratio (HR) with their associated 95% CI and p-values. The analysis was performed considering both single-tooth elements and patients. Moreover, Kaplan-Meier curves were plotted to graphically show the differences of each variable in the survival of the restoration. Log-rank test results between groups were reported. Statistical significance was set at p < 0.05.

## Results

In total, 168 restorations were evaluated in 90 patients (mean age 28.6 ± 11.8 years; 38 male and 52 female) with an average follow-up period of 37.9 (± 22.9) months. A total of 132 restorations were performed on vital teeth, and 36 were performed on ETT. A total of 128 Class-IV and 40 Class-III restorations were performed. In 89 restorations, a three-step E&R adhesive was applied (14 Class-III and 75 Class-IV), while 79 were placed with a universal adhesive (26 Class-III and 53 Class-IV). That is, 32.9% of the Class-III restorations were performed with the E&R adhesive, but 15.7% of them with a universal adhesive; this represents a statistically significant difference (p = 0.0091).

**Fig 3:**
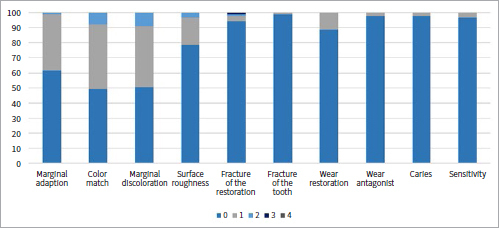
Distribution of USPHS criteria scores (0-4) for the three-step etch-and-rinse group.

**Fig 4:**
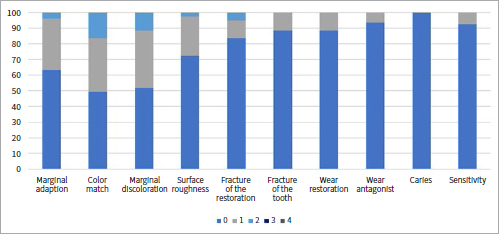
Distribution of USPHS criteria scores (0-4) for the universal adhesive in etch-and-rinse mode group.

**Fig 5:**
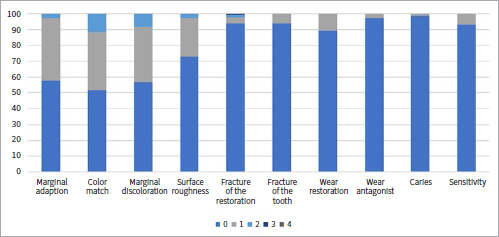
Distribution of USPHS criteria scores (0-4) for the vital teeth group.

**Fig 6:**
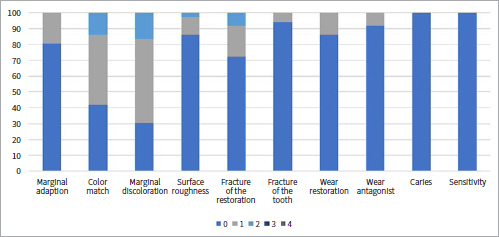
Distribution of USPHS criteria scores (0-4) for the endodontically treated teeth group.

**Fig 7:**
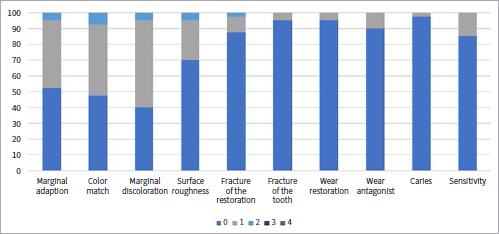
Distribution of USPHS criteria scores (0-4) for the class III cavity group.

**Fig 8:**
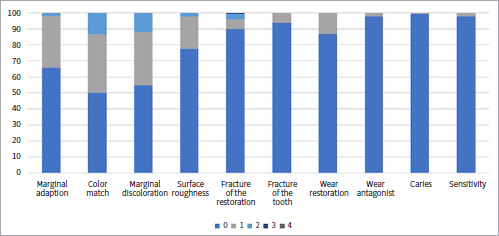
Distribution of USPHS criteria scores (0-4) for theclass IV cavity group.

**Fig 9:**
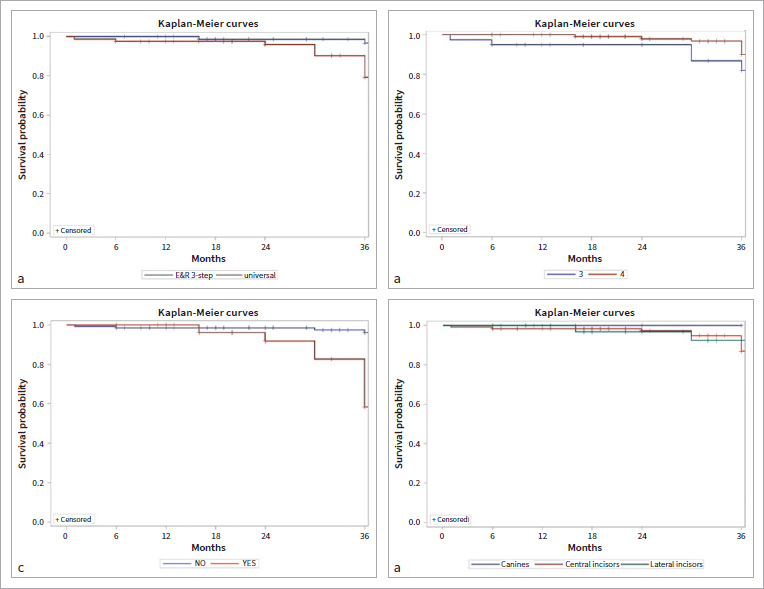
Kaplan-Meier curves: a) adhesive types, p = 0.0377; b) classes, p = 0.2666; c) endodontic treatment, p = 0.0001; d) tooth element, p = 0.4900.

Table 1 shows the collected data and the number of failures and survivals of restorations in the different groups and subgroups. Figures 3 to 9 present the qualitative evaluation at follow-up using USPHS criteria for those restorations still in situ in three-step E&R groups (Fig 3), for the universal adhesive in the E&R group (Fig 4), vital teeth (Fig 5), ETT (Fig 6), Class-III cavities (Fig 7), and Class-IV cavities. A general successful result was observed for all groups. Restoration debonding – classified as absolute failure – was recorded in 1.1% of the cases in the three-step E&R group, in 0.8% of the cases in the vital teeth group, and 0.8% of the cases in the Class-IV cavity group.

**Table 1:** Number of cases (N) considered as failed (F) and survived (SR) for the different groups and subgroups

			
**Total restorations** **N = 168**	**E&R three-step** **(N = 89)** F = 2 SR = 15	**Class III** **(N = 14)** F = 0 SR = 2	**Vital teeth** **(N = 14)** F = 0 SR = 2
			**ETT** **(N = 0)** F = 0 SR = 0
		**Class IV** **(N = 75)** F = 2 SR = 13	**Vital teeth** **(N = 58)** F =1 SR = 10
			**ETT** **(N = 17)** F = 1 SR =3
	**Universal** **(N = 79)** F = 4 SR = 6	**Class III** **(N = 26)** F = 1 SR = 6	**Vital teeth** **(N= 21)** F = 1 SR = 4
			**ETT** **(N = 5)** F = 0 SR = 2
		**Class IV** **(N = 53)** F = 3 SR = 10	**Vital teeth** **(N = 39)** F = 1 SR = 7
			**ETT** **(N = 14)** F = 3 SR = 2

In Table 2, the details of the failures are shown. Marginal adaptation (p = 0.2565), color matching (p = 0.0861), marginal discoloration (p = 0.6061), surface roughness (p = 0.7494), wear of restoration (p = 0.9745), and wear of antagonist (p = 0.1863) showed no differences between the three-step E&R and universal adhesives.

**Table 2:** Number and percentage of failures for the two adhesive systems by USPHS criteria

	E&R three-step N (%)	Universal N (%)	p-value
Failure in marginal adaptation	1 (1.12)	3 (3.8)	0.2565
Failure in color matching	7 (7.87)	13 (16.46)	0.0861
Failure in marginal discoloration	8 (8.99)	9 (11.39)	0.6061
Failure in surface roughness	3 (3.37)	2 (2.53)	0.7494
Fracture restoration	5 (5.62)	13 (16.46)	0.0234
Fracture tooth	0 (0)	0 (0)	–
Failure in wear restoration	10 (11.24)	9 (11.39)	0.9745
Failure in wear antagonist	2 (2.25)	5 (6.33)	0.1863
Caries	2 (2.25)	0 (0)	0.1801
Postoperative sensitivity	0 (0)	0 (0)	–

Restorations placed with universal adhesives showed significantly more failures by fracture of the restoration (p = 0.0234). Marginal adaptation never occurred in Class-IV cavities (p = 0.0199), while in Class-III, there were more failures due to wear of the antagonist (p = 0.0206). No statistical differences were noticed considering caries (p = 0.1801) and postoperative sensitivity.

To understand which factors were involved in the failure of restorations in three years, a Cox regression analysis was performed. The adhesive employed (universal vs E&R, HR = 2.8, 1.0–8.2 95% CI, p = 0.0495) and endodontic treatment (yes vs no, HR = 5.2, 2.0–13.4 95% CI, p = 0.0006) are statistically significant in the model, while tooth element (p = 0.2922) and vitality of the tooth (p = 0.2805) were not found to be factors leading to fracture of the restoration.

The best performance during the follow-up of the E&R adhesive can also be seen in the Kaplan-Meier plot in Fig 9a, where the “universal” curve is significantly lower than the E&R curve (p = 0.0377). In Fig 9b, the trend of the different classes is shown: no statistically significant difference was recorded between Class-III and Class-IV (p = 0.2666). Figure 9c shows that patients who had undergone endodontic treatment had a significantly worse outcome (p = 0.0001). No statistically significant differences were evident between tooth elements (p = 0.49; Fig 9d).

The same Cox model was used considering a failure of at least one of the outcomes as an event (Table 3). The only significant factor was tooth type: restorations of canines failed more frequently (HR = 3.8, 95% CI 1.4–10.1, p = 0.0062). However, it should be noted that there were relatively few canines.

**Table 3:** Cox models HR estimation from Cox regression model with 95 % CI and p-values

Failure in fracture restoration		
Parameter	HR (95%CI)	P value
Adhesive: Universal vs E&R	2.884 (1.002–8.297)	0.0495
Class: III vs IV	1.808 (0.617–5.299)	0.2805
Endodontic treatment: yes vs no	5.204 (2.02–13.403)	0.0006
Canines vs lateral	–	0.9934
Central vs lateral	2.206 (0.506–9.611)	0.2922
Any failure		
Parameter	HR (95%CI)	p-value
Adhesive: Universal vs e&r	1.101 (0.771–1.571)	0.597
Class: III vs IV	1.363 (0.913–2.034)	0.1294
Endodontic treatment: yes vs no	1.478 (0.979–2.23)	0.063
Canines vs lateral	3.852 (1.466–10.12)	0.0062
Central vs lateral	0.9 (0.597–1.358)	0.6172

## Discussion

The first aim of the present study was to analyze the clinical longevity of direct composite restorations to restore Class-III and Class-IV cavities with a three-step E&R or universal adhesive employed using an E&R protocol. A general successful result was observed for both adhesives tested after a mean follow-up period of 37.9 (± 22.9) months, despite the fact that composite restorations made with universal adhesives showed significantly more failures due to fracture (16.46%, p = 0.0234) compared to the three-step E&R adhesive (5.62%). Therefore, the first null hypothesis was accepted, since composite fracture is an event that could not be directly related to the adhesive. Different factors can be implicated in the fracture of anterior restorations, such as masticatory load and incisal stress^12^ (especially in Class-IV restorations), parafunctional activity,^31^ and the thickness of the restoration.^7,17^ The results of the present study suggest that anterior restorations placed using a universal adhesive in E&R mode seem to be more prone to fracture over time. However, as previously mentioned, this result should be interpreted with caution. The adhesive does not directly affect the partial fracture of a composite restoration; rather, it may be involved with marginal discoloration, secondary caries, post-operative sensitivity, marginal fracture, or debonding. However, all restoration fractures observed in the universal adhesive group (16.46%) consisted of composite chipping, which did not require composite replacement, and were therefore were classified as SR. On the other hand, the absolute number of restoration fractures in the three-step E&R group was lower (5.62%), but one restoration debonding was observed. Nonetheless, no statistical differences were observed when considering parameters directly involved with the adhesives tested, such as marginal adaptation, marginal discoloration, or post-operative sensitivity, which showed similar clinical performances.

One of the weaknesses inherent in the older simplified adhesives, such as two-step E&R or one-step SE, which may influence the clinical success of a composite restoration over the years was the incorporation of hydrophilic and acidic resin monomers.^48^ The presence of hydrophilic resin is correlated with an increase in permeability to fluid movements and, consequently, to an increase in water sorption, which can lead to nanoleakage.^46,47^ Water sorption also leads to a decrease in the elastic module and a reduction in bond strength.^24^ This chemical factor, together with mechanical factors such as occlusal load and expansion and contraction stress due to thermal changes,^20^ may influence the long-term mechanical behavior of the composite. All of this can explain how simplified adhesives can ensure better clinical performance when used in anterior tooth restorations, where the amount of exposed dentin and occlusal loads are lower than in posterior teeth.

To date, the ability of an enamel-dentin adhesive to seal the dentin is one of key variables influencing the service life of a restoration in the oral cavity. Nevertheless, different adhesives may influence the thickness and homogeneity of the hybrid layer, which are directly correlated with bond stability.^3^ Concerning universal adhesives, Fujiwara et al^19^ investigated in vitro the effect of the number of adhesive layer applications on the mechanical properties of the hybrid layer and concluded that double-layer application of universal adhesives may enhance both initial and long-term bond stability.

The second aim of the present study was to investigate differences in clinical performance between vital and non-vital teeth. The results show that ETT were more prone to fractures (p = 0.0006); thus, the second null hypothesis was rejected. However, it must be borne in mind that the two groups contained different numbers of teeth (vital teeth n=132, ETT n= 36), so that any conclusions must be made with caution. Consequently, further studies are required to validate the present result. In the case of an ETT, the treatment protocol may be influenced by the amount of residual dental structure.^15^ Among different treatment options, such as full crown^44^ or indirect restoration,^16^ direct restorations represent the most conservative approach, maximizing the preservation of sound tooth tissue.

On the other hand, the remaining tooth structure after cavity debridement is a key factor in the stress resistance of ETT in anterior and posterior teeth. As shown in a recent study, the loss of one or two marginal ridges, which represent the anatomical portion in anterior teeth which resists transversal loads, is immediately correlated with an increased interfacial gap, the first step of mechanical degradation that could lead to tooth fracture.^6^ Therefore, since all non-vital teeth treated in the present study presented a large amount of sound tooth structure, a direct approach was followed, as in several other studies (ETT).^27,42^ However, the obtained data showed that having an ETT may be an important risk factor for the longevity of a direct anterior restoration. This is supported by Coelho-de-Souza et al,^5^ who report that direct anterior veneers on ETT have double the risk of failure compared to vital teeth.

The literature contains many in-vitro studies on the application of universal adhesives,^34,38,53^ while long-term in-vivo trials are still relatively rare, particularly for anterior teeth. To the authors’ knowledge, no other studies have compared these two adhesives in direct anterior restorations. Currently, data available from previous studies is limited to the treatment of posterior teeth and NCCL. In 2019, Carvalho et al^4^ investigated the influence of different application protocols (E&R and SE) of universal adhesives in the treatment of Class-I and Class-II direct restorations over a follow-up period of 15.8 ± 2.7 months, showing no influence of the application protocols on the clinical behavior of composite restorations. More recently, Yazici et al^55^ showed that after 48 months of follow-up, the E&R approach with a universal adhesive appeared to be advantageous in terms of marginal discoloration, while other authors found that the SE approach with the same bonding system did not negatively affect clinical success when employed with bulk-fill resin restorations. In 2020, Matos et al^14^ showed better clinical behavior after five years of follow-up of a universal adhesive when applied in E&R mode instead of an SE strategy in the treatment of NCCL. This was in agreement with other studies that evaluated other universal adhesives but reached the same conclusion.^9,18^

## Conclusion

This retrospective clinical evaluation showed that both universal and three-step E&R adhesives used for direct composite restorations on anterior teeth are a good approach in the mid term. Furthermore, tooth vitality seems to be fundamental for a good longer-term prognosis of direct composite restorations in anterior teeth. Nevertheless, in terms of composite failures and the survival rate of restorations, long-term follow-ups are still necessary to confirm the advantages of universal adhesives over multistep adhesives.

## Clinical relevance

In direct class III or class IV restorations of anterior teeth, universal adhesives in E&R mode show good mid-term durability and may be a suitable alternative to traditional three-step E&R adhesives.
